# Problems on the back of an envelope

**DOI:** 10.7554/eLife.19569

**Published:** 2016-09-08

**Authors:** Polina Anikeeva, Alan Jasanoff

**Affiliations:** 1 Department of Materials Science and Engineering and the Research Laboratory of Electronics, Massachusetts Institute of Technology, Cambridge, United Statesanikeeva@mit.edu; 2 Department of Biological Engineering, the Department of Brain and Cognitive Sciences, and the Department of Nuclear Science and Engineering, Massachusetts Institute of Technology, Cambridge, United Statesjasanoff@mit.edu

**Keywords:** magnetogenetics, physical plausibility, magnetoreception, magnetic control, None

## Abstract

Claims that magnetic fields can be used to manipulate biological systems contradict some basic laws of physics.

**Related research article** Meister M. 2016. Physical limits to magnetogenetics. *eLife* **5**:e17210. doi: 10.7554/eLife.17210

Many biophysicists have in their library a little book by Howard Berg called *Random Walks in Biology*. In this book Berg applies principles one learns in an introductory physical chemistry class to analyze processes ranging from the migration of proteins on gels to the motility of whole living cells (Berg, 1993). In addition to exploring a specific set of phenomena, the book promotes the ethos of using simple models and calculations – often so simple that they could be performed on "the back of the envelope" – to bring order to otherwise messy biological systems. This ethos gives life scientists a quantitative alternative to the qualitative empiricism that is found in many fields of modern biology.

Now, in *eLife*, Markus Meister of Caltech reports how the results of such calculations have led him to question a number of recent papers about the use of magnetic fields to manipulate proteins, cells and organisms (Meister, 2016). Because biological tissue normally interacts only weakly with magnetic fields, it might be possible to use magnetically-active molecules that interface with biological systems to remotely control specific physiological processes. Given the wealth of research made possible by optogenetics – in which light is used to control cells that have been genetically modified to make them sensitive to light (Adamantidis et al., 2015) – one can only imagine the possibilities afforded by an analogous “magnetogenetic” approach. The papers that Meister critiques all claim to harness iron-binding proteins to realize various aspects of magnetogenetics. However, using arguments from elementary physics, he shows that key results reported in these articles cannot be explained by the magnetic phenomena the authors say they have exploited (Figure 1).

**Figure 1.**
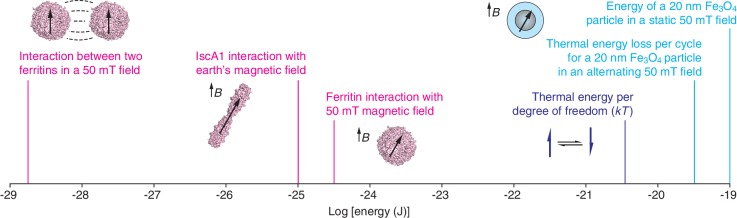
Molecular-scale magnetic interaction energies. The energies associated with various magnetogenetic components, as estimated optimistically by Meister, are labeled in magenta. Each is orders of magnitude smaller than the thermal energy per degree of freedom at room temperature (dark blue). In contrast, based on our calculations, interactions involving magnetite particles (Fe_3_O_4_; light blue) can exceed the thermal energy (*kT*) at room temperature. These calculations assume that the magnetization of magnetite is 480 emu/cm^3^ and that the specific loss power for an individual particle is 24 fW. Note that the x-axis is logarithmic. The strength of the earth's magnetic field varies between about 25 and 65 microtesla at the earth's surface. mT: millitesla; B: magnetic field; k: Boltzmann’s constant; T: temperature.

In the first paper, Can Xie of Peking University and co-workers report that Isca1 – an iron-sulfur cluster protein that is found in Drosophila – acts like a compass needle and aligns with the Earth’s magnetic field (Qin et al., 2015). Such a "biocompass" could be a potential molecular building block for magnetogenetics. However, Meister’s calculations show that Isca1 (Cózar-Castellano et al., 2004) is not capable of acting like a compass needle. The iron content of Isca1 was proposed as a basis for magnetic effects. Certain configurations of iron give rise to molecular-scale magnetic dipoles, which interact with magnetic fields in roughly the same way a miniscule bar magnet would. But Meister points out that the smallest iron-containing species with dipoles strong enough to spontaneously orient in magnetic fields at room temperature are mineral particles with diameters of ~20 nm, each of which contains hundreds of thousands of iron atoms in very close proximity to each other. Both the quantity and closeness of the iron atoms in such nanoparticles are necessary in order for the magnetic contributions of the individual atoms to cooperate with each other sufficiently to override the randomizing effects of thermal energy (Cullity and Graham, 2008), and the 40 iron atoms in Isca1 are too few and too far apart to achieve this. Even if the atoms were close enough, Meister calculates that the interaction between the protein and the earth's magnetic field would be much too weak (by a factor of about 10^5^) to overcome thermal fluctuations at room temperature.

Rather than searching for naturally occurring building blocks for magnetogenetics, other researchers have used genetic techniques to produce hybrid protein assemblies that contain the iron storage protein ferritin conjugated to an ion channel. Ali Güler of the University of Virginia and co-workers (Wheeler et al., 2016) studied neurons in which the ferritin was conjugated to an ion channel called TRPV4 that is activated by mechanical force (Liedtke, 2008). Güler and co-workers wanted to use magnetic fields to rotate or pull on the ferritin components, thus exerting a force that could be used to trigger the opening of the ion channel. The researchers reported that they observed electrophysiological and behavioral changes when they applied magnetic field gradients to various systems, including in vitro systems, brain slices and freely moving mice. However, Meister’s analysis indicates that the magnetic field conditions used in the study could produce forces that were, at most, nine orders of magnitude lower than the forces needed to open known mechanically-sensitive ion channels. Indeed, the forces are too weak (by several orders of magnitude) to overcome random thermal fluctuations at room temperature, and are thus unlikely to mediate magnetogenetics.

Jonathan Dordick (Rensselaer Polytechnic Institute), Jeffrey Friedman (Rockefeller University) and co-workers (Stanley et al., 2015) studied insulin-secreting cells in which the ferritin was conjugated to a temperature-sensitive ion channel called TRPV1 (Caterina et al., 1997). The basic idea was that applying an oscillating magnetic field would heat the ferritin and that the resulting increase in temperature would open the ion channel, triggering insulin release. The approach is inspired by a similar strategy, called magnetic hyperthermia, that uses oscillating fields to heat much more strongly magnetic particles, and can be used to treat cancer (Pankhurst et al., 2009). Stanley et al. reported that ion channels in mice could be activated by oscillating magnetic fields. However, Meister estimated the specific loss power *–* the amount of heat dissipated by a particle per gram of iron *–* for the ferritin nanoparticles (Fantechi et al., 2014) used in the experiments and found that it was too low to support a magnetogenetic approach.

Where does magnetogenetics go from here? Two ways forward seem plausible. The first is to continue efforts to control biological systems using magnetic species that are far more potent than Isca1 or ferritin. Indeed, two groups have already demonstrated that nanoparticles with magnetic moments ~1000 times higher than those of ferritin can be used to manipulate neural activity (Huang et al., 2010; Chen et al., 2015). Using magnetic fields to heat such nanoparticles in close proximity to temperature-sensitive ion channels should lead to a physically practical form of the technique proposed by Stanley *et al.* Another challenge is to deliver these nanoparticles to where they are needed: in the future, it might be possible to do this by repurposing the genetic machinery that enables certain bacterial and vertebrate species to produce their own magnetite (Johnsen and Lohmann, 2005). A second way forward might involve a critical re-examination of the experiments performed by Wheeler et al. and Stanley et al. If their cellular-level findings prove reproducible, the true mechanism that explains them might even fulfill some of the promise that magnetogenetics seemed to offer before it met the back of Professor Meister’s envelope.
